# Optimal treatment of ceftazidime-avibactam and aztreonam-avibactam against bloodstream infections or lower respiratory tract infections caused by extensively drug-resistant or pan drug-resistant (XDR/PDR) *Pseudomonas aeruginosa*

**DOI:** 10.3389/fcimb.2023.1023948

**Published:** 2023-06-28

**Authors:** Yixin Kang, Lu Xie, Jiyong Yang, Junchang Cui

**Affiliations:** ^1^ Department of Respiratory Diseases, The first Medical Center, Chinese People’s Liberation Army General Hospital, Beijing, China; ^2^ Medical School of Chinese People’s Liberation Army (PLA), Beijing, China; ^3^ Research Center for Micro-Ecological Agent Engineering and Technology of Guangdong Province, Guangzhou, China; ^4^ Department of Laboratory, The First Medical Center, Chinese People’s Liberation Army General Hospital, Beijing, China; ^5^ College of Pulmonary & Critical Care Medicine, 8th Medical Center, Chinese People’s Liberation Army General Hospital, Beijing, China

**Keywords:** extensively drug-resistant *Pseudomonas aeruginosa*, pan drug-resistant *Pseudomonas aeruginosa*, ceftazidime-avibactam, 173 aztreonam-avibactam, whole-genome sequencing

## Abstract

**Objective:**

To evaluate the efficacy of ceftazidime-avibactam (CZA) and aztreonam-avibactam (AZA) against bloodstream infections (BSIs) or lower respiratory tract infections (LRTIs) – caused by extensive drug-resistant or pan drug-resistant (XDR/PDR) *Pseudomonas aeruginosa.*

**Method:**

The two-fold dilution method was used to determine the minimum inhibitory concentrations (MICs) of CZA/AZA against XDR/PDR *P. aeruginosa*. Whole-genome sequencing was used to analyze the resistance determinants of each isolate. Monte Carlo simulations (MCSs) were used to evaluate the probability of target attainment (PTA) and the cumulative fraction of response (CFR) of each CZA/AZA dosing regimen *via* traditional infusion (TI)/optimized two-step-administration therapy (OTAT).

**Results:**

We found that XDR/PDR P. aeruginosa may carry some rare MBLs (e.g.: IND-6, SLB-1, THIN-B). *P. aeruginosa* isolates producing IMP-45, VIM-1, or VIM-2 were inhibited by AZA at a concentration of 2 to 8 mg/L. All isolates producing IND-6 plus other serine β-lactamases were high-level resistant to CZA/AZA (MICs >64 mg/L). All simulated dosing regimens of CZA/AZA against BSIs-causing XDR/PDR *P. aeruginosa* achieved 100% PTA when the MIC was ≤32 mg/L.

**Conclusion:**

AZA has been considered as an option for the treatment of infections caused by XDR/PDR *P. aeruginosa* producing IMP-45, VIM-1, or VIM-2. OTAT with sufficient pharmacodynamic exposure may be an optimal treatment option for XDR/PDR *P. aeruginosa* with a high-level MIC of CZA/AZA.

## 1 Introduction

In the last decade, *P. aeruginosa* has spread widely throughout the world, posing a significant burden to the daily work of physicians and a serious threat to the lives of patients. *P. aeruginosa* displays resistance to various antibiotics, making treatment challenging (Horcajada et al., 2019). The Centers for Disease Control and Prevention (CDC) have defined multidrug resistant (MDR) and extensively drug-resistant (XDR) *P. aeruginosa* as a serious threat level (Centers for Disease Control and Prevention).

Ceftazidime/avibactam (CZA) is a novel β-lactam/β-lactamase inhibitor (BL/BLIs). And it was approved by the Chinese National Medical Products Administration (CNMPA) in 2019 for the treatment of complicated intra-abdominal infections (cIAIs), hospital-acquired pneumonia (HAP), and ventilator-associated pneumonia (VAP), and in adult patients with limited treatment options for infections caused by the following gram-negative bacteria sensitive to this product: *Klebsiella pneumoniae*, *Enterobacter cloacae*, *Escherichia coli*, *Acinetobacter chimaerae*, and *P. aeruginosa* (Product information a). CZA has good safety and was regarded as a vital treatment option for *P. aeruginosa* infections. CZA showed good capabilities against *P. aeruginosa* with a sensitivity rate ranging from 76.2% to 97.8% (Sader et al., 2017a). In terms of clinical efficacy, the clinical success rate of CZA on *P. aeruginosa* infections ranged from 64.3% to 90.6% (Mazuski et al., 2016; Torres et al., 2018).

Aztreonam (ATM) was the first monobactam antibiotic to be used in clinical therapy. It was approved by the U.S. Food and Drug Administration (FDA) in 1986 for treatment of various infections caused by sensitive aerobic gram-negative bacteria. ATM was stable to hydrolysis by Ambler class B Metallo-β-lactamases (MBLs) (Yong et al., 2009). However, MBLs-producing bacteria also produce other types of *β*-lactamases ((i.e., ESBLs, AmpC enzymes) against which ATM is ineffective. Avibactam (AVI) is a *β*-lactamase inhibitor with a wide enzyme inhibition spectrum, including Ambler class A (KPC, TEM), class C (AmpC), and class D (OXA-48 type) *β*-lactamase. The combination of ATM and AVI can potentially inhibit MBL-producing bacteria (Bhatnagar et al., 2021).

We performed antimicrobial susceptibility testing (AST) on XDR/PDR *P. aeruginosa* in this study. Whole-genome sequencing and bioinformatic analysis were used to identify resistance genes of each isolate. Besides, we combine the population pharmacokinetic parameters (PPKs) of CZA/AZA with minimum inhibitory concentration (MIC) distribution of XDR/PDR *P. aeruginosa* to evaluate the efficacy of various dosing regimens. Therefore, the objectives of our work are as follows. Firstly, our work aims to compare the *in vitro* activity of CZA and AZA against XDR/PDR *P. aeruginosa*. Secondly, our team wants to evaluate the relationship between resistance genes and CZA/AZA sensitivity rates of XDR/PDR *P. aeruginosa*. Thirdly, we assess the efficacy of CZA and AZA for the treatment of critically ill patients with BSIs/LRTIs caused by XDR/PDR *P. aeruginosa*.

## 2 Materials and methods

### 2.1 Bacterial strains and antimicrobial agents

We collected 67 *P. aeruginosa* from critically ill patients admitted to the First Medical Centre of Chinese PLA General Hospital from January 2016 to November 2021. A total of 10 *P. aeruginosa* strains were categorized as XDR according to CLSI criteria. Moreover, 57 *P. aeruginosa* strains were categorized as PDR (Abbey and Deak, 2019). All *P. aeruginosa* were identified by VITEK^®^2 system (bioMérieux, Marcy-l’Étoile, France). Ceftazidime, avibactam, and aztreonam standards were purchased from MedChemExpress. The resistance rates of XDR/PDR *Pseudomonas aeruginosa* to cefoperazone-sulbactam, imipenem, ciprofloxacin, piperacillin-tazobactam, ceftazidime, levofloxacin, meropenem, tobramycin, amikacin and gentamicin were 89.6%, 100%, 89.6%, 100%, 97%, 89.6%, 100%, 97.1%, 100%, 100%, respectively.

### 2.2 Antimicrobial susceptibility tests

We used the broth two-fold dilution method to determine the minimum inhibitory concentrations (MICs) of CZA/AZA against XDR/PDR *P. aeruginosa*. A fixed concentration of AVI at 4 mg/L, 8mg/L, and 16mg/L combined with 2-fold diluted CAZ and ATM were used in ASTs. The quality control strains of our tests were *E. coli* ATCC 25922 and *P. aeruginosa* ATCC 27853. The MICs are defined as the lowest concentration of antibiotics that inhibits the growth of bacteria. The definition of MIC_50_ is a drug concentration that inhibits the growth of bacteria by 50%. Similarly, MIC_90_ is a drug concentration inhibiting 90% of bacterial growth. MIC distributions of CZA/AZA against *P. aeruginosa* were represented by cumulative inhibition rates (CIRs). Besides, all experiments were conducted three times following Clinical and Laboratory Standards Institute (CLSI) standards.

### 2.3 Whole-genome sequencing

67 *P. aeruginosa* were subjected to whole-genome sequencing (WGS) using Illumina MiSeq short-read sequencing (Illumina, San Diego, CA, USA). Sequenced isolates were evaluated using FASTQC, version 0.11.6, and MultiQC, version 1.6. Trimmomatic, version 0.39, removed adapters and trimmed low-quality paired end reads. Comprehensive Antibiotic Resistance Database v.1.2.0 (McMaster University, Hamilton, Ontario) was used to identify drug resistance genes in the strains.

### 2.4 Pharmacokinetic/pharmacodynamics modeling

Population pharmacokinetic (PPK) parameters of CZA and AZA were obtained from previously published articles (Vinks et al., 2007; Stein et al., 2019; Cornely et al., 2020). CAZ is a time-dependent antibiotic, %50*fT* > *MIC* is the best indicator for assessing BSIs. Besides, %50*fT* > 5 × *MIC* is the best indicator for assessing LRTIs. When combined with CAZ, %50*fT* > *CT* of 1 mg/L was considered the *Pharmacokinetic (PK)/Pharmacodynamics (PD)* target of avibactam. PK/PD targets of ATM for BSIs was %60*fT* > *MIC*. And PK/PD targets of ATM for LRTIs were %60*fT* > 5 × *MIC*. As for avibactam, %50*fT* > *CT* of 2.5 mg/L was considered appropriate for guiding dosage selection for AZA (Nichols et al., 2018). Optimized two-step administration therapy (OTAT) refers to a rapid injection (0.5h) of a loading dose in the first step and a continuous infusion (2h) in the second step to maintain adequate drug exposure. The %*fT* > *n* × *MIC* equation was based on previous studies (Eguchi et al., 2010; Schaumburg et al., 2019; Song et al., 2019).

### 2.5 Monte carlo simulations

We conducted 10000-patient Monte Carlo simulations (MCSs) using Oracle Crystal Ball version.11.1.24. PK parameters (V_d_, CL, t_1/2_) followed a log-normal distribution. All simulated dosing regimens *via* traditional infusion (TI)/optimized two-step-administration therapy (OTAT) were listed in **Table 1**. The definition of probability of target attainment (PTA) was the probability of reaching the PK/PD target at different MICs. The equation for cumulative fraction of response (CFR) is $CFR=\sum_{i=1}^{n}PTA(MICi)\times p(MICi)$ . *MICi* means each MIC value. *p*(*MICi)* means the percentage of each MIC value. A CFR ≥ 90% is adequate PD exposure for this dosing regimen.

**Table 1:** The susceptibility rate of CZA against XDR/PDR *P. aeruginosa* and the MIC50 and MIC90 of AZA against XDR/PDR *P. aeruginosa*.

Isolates	CZA				AZA		
	MIC_50_ (mg/L)	MIC_90_ (mg/L)	MIC range (mg/L)	S (%)	MIC_50_ (mg/L)	MIC_90_ (mg/L)	MIC range (mg/L)
XDR-PA (10)	16	>64	4–>64	40.0	16	64	2–>64
PDR-PA (57)	32	>64	4–>64	19.3	>64	>64	8–>64

Since the concentration of avibactam is fixed at 4 mg/L in clinical practice, only the MIC of avibactam at 4 mg/L is indicated in this table.

## 3 Results

### 3.1 Resistance genes

From the predicted results, antibiotic efflux accounted for approximately 75%, previous studies have also shown that efflux pumps are a mechanism for the acquisition of drug resistance in this organism (Horcajada et al., 2019), and furthermore the presence of antibiotic inactivation, such as OXA beta-lactamase. This study further discusses the clinical potential of beta-lactamase inhibitors in combination with other drugs for the treatment of this bacterial infection.

### 3.2 Sensitivity tests

**Figure 1:**
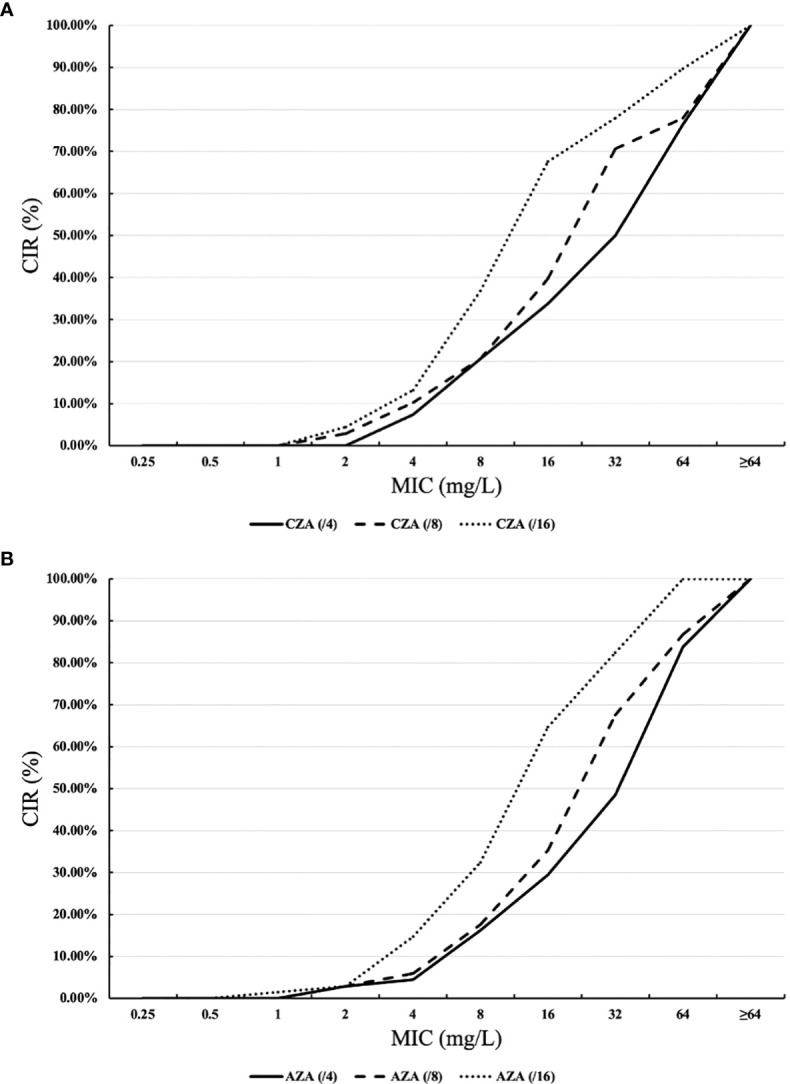
Cumulative inhibition ratios (CIRs) of ceftazidime-avibactam (CAZ) and aztreonam (ATM) against XDR/PDR *P. aeruginosa.* **(A)** ceftazidime +avibactam, **(B)** aztreonam +avibactam. MIC: minimum inhibitory concentration. The horizontal axis of (Part label **A**) represents the MICs of ceftazidime combined with avibactam against XDR/PDR *P. aeruginosa* at avibactam concentrations of 4 mg/L, 8 mg/L, and 16 mg/L. The vertical axis of (Part label **A**) shows the CIRs of ceftazidime in combination with avibactam against XDR/PDR *P. aeruginosa* at avibactam concentrations of 4 mg/L, 8 mg/L, and 16 mg/L. Similarly, the horizontal axis of (Part label **B**) represents the MICs of aztreonam in combination with avibactam against XDR/PDR *P. aeruginosa* at avibactam concentrations of 4 mg/L, 8 mg/L, and 16 mg/L. The vertical axis of (Part label **B**) shows the CIRs of aztreonam in combination with avibactam against XDR/PDR *P. aeruginosa* at avibactam concentrations of 4 mg/L, 8 mg/L, and 16 mg/L.

**Figure 1** shows the cumulative inhibition ratios (CIRs) of ceftazidime (CAZ) and aztreonam (ATM) with increased avibactam concentration against XDR/PDR *P. aeruginosa.* As the concentration of avibactam increased, the CIRs of XDR/PDR *P. aeruginosa* by ceftazidime and aztreonam increased. As shown in **Table 1**, the susceptibility rate of CZA against 57 PDR *P. aeruginosa* and 10 XDR *P. aeruginosa* was 19.3% and 40%, respectively. Besides, the MIC_50_ and MIC_90_ of CZA against PDR *P. aeruginosa* were 32mg/L and >64mg/L, respectively. The MIC_50_ and MIC_90_ of CZA against XDR *P. aeruginosa* were 16mg/L and >64mg/L, respectively. CLSI has not published the breakpoint of AZA. When the concentration of avibactam was 4mg/L, AST showed that the MIC_50_ and MIC_90_ of AZA against PDR *P. aeruginosa* were 64mg/L and >64mg/L, respectively. Similarly, when the concentration of avibactam was 4mg/L, the MIC_50_ and MIC_90_ of AZA against XDR *P. aeruginosa* were 16mg/L and >64mg/L, respectively.

### 3.3 Comparative MICs of CZA and AZA

The comparative MICs (mg/L) of CZA and AZA against 67 XDR/PDR *P. aeruginosa* positive for the OXA gene (with or without other *β*-lactamase enzymes) were listed in **Table 2**. Most XDR/PDR *P. aeruginosa* with an OXA-101 also produced OXA-850. CZA was as effective as AZA against these isolates. For isolates with an OXA gene plus IMP-45, VIM-1, or VIM-2, AZA was much more potent than CZA against these isolates, with all isolates being inhibited by a concentration of 8 mg/L. For isolates with an IND-6 plus more serine β-lactamases, all isolates produced CZA/AZA MICs of >64 mg/L. For XDR/PDR *P. aeruginosa* with other genotypes, the efficacy of CZA against these isolates did not differ significantly from that of AZA.

**Table 2:** Comparative MICs (mg/L) range for CZA and AZA against 67 XDR/PDR *P. aeruginosa* positive for OXA gene alone and one or more additional *β*-lactamase genes.

Group (n)	MICs (mg/L) range for	
	CZA	AZA
OXA-1 + OXA-50 + IMP-45 (2)	>64	2–16
OXA-1 + OXA-488 + OXA-573 + VIM-1 + IMP-45 (1)	>64	2
OXA-7 + OXA-50 + OXA-101 (1)	4	8
OXA-10 + OXA-50 + VIM-2 (1)	>64	8
OXA-17 + OXA-129 + OXA-488 (2)	32–64	32–64
OXA-50 (5)	4–64	4–32
OXA-50 + OXA-101 + OXA-573 (1)	64	64
OXA-50 + OXA-246 + PEDO-3 (1)	64	64
OXA-50 + OXA-573 (2)	8–16	16–32
OXA-101 + OXA-246 + OXA-573 + OXA-846 (1)	64	64
OXA-101 + OXA-488 + OXA-573 (1)	8	16
OXA-101 + OXA-573 + OXA-850 + THIN-B (1)	16	64
OXA-101 + OXA-573 + OXA-850 (1)	16	32
OXA-101 + OXA-850 (22)	8–>64	16–64
OXA-101 + OXA-850 + IND-6 (8)	>64	>64
OXA-101 + OXA-850 + SLB-1 (1)	64	>64
OXA-129 + OXA-488 (1)	64	64
OXA-246 + OXA-486 + OXA-573 + KPC-2 (3)	8–16	8–16
OXA-246 + OXA-573 + OXA-846 (2)	64–>64	64–>64
OXA-488 + OXA-573 (1)	4	16
OXA-488 (2)	8	16
OXA-573 + OXA-846 (1)	16	32
OXA-846 (2)	4–16	8–32
OXA-850 (4)	8–64	8–64

### 3.4 Probability of target attainment

#### 3.4.1 Probability of target attainment of BSIs

The probability of target attainment (PTA) of each simulated dosing regimen for BSIs was listed in **Table 3A**. All dosing regimens of CZA/AZA achieved a PTA of 100% when the MIC was ≤32 mg/L. When the MIC was 64 mg/L, CZA 2.5g q8h declined to a PTA of 88.6%. When the MIC was >64 mg/L, CZA 2.5g q8h, 2.5g q6h, 4g q8h, 4g q6h, 1.25g (0.5h) +1.25g (2h) q8h, 1.25g (0.5h) +1.25g (2h) q6h, 0.675g (0.5h) +0.675g (2h) q8h, 0.675g (0.5h) +0.675g (2h) q8h, 0.675g (0.5h) +0.675g (2h) q6h achieved a PTA of 0, 5.48%, 2.78%, 88.85%, 93.21%, 96.05%, 80.34% and 80.03%, respectively. When the MIC was 64 mg/L, AZA 2.5g q8h, 0.675g (0.5h) +0.675g (2h) q8h and 0.675g (0.5h) +0.675g (2h) q6h achieved a PTA of 89.49%, 93.77% and 99.83%, respectively. When the MIC was >64 mg/L, AZA 2.5g q8h, 2.5g q6h, 4g q8h, 4g q6h, 1.25g (0.5h) +1.25g (2h) q8h, 1.25g (0.5h) +1.25g (2h) q6h, 0.675g (0.5h) +0.675g (2h) q8h, 0.675g (0.5h) +0.675g (2h) q8h, 0.675g (0.5h) +0.675g (2h) q6h achieved a PTA of 0, 75.75%, 14.15%, 91.78%, 94.35%, 99.77%, 7.43%, 14.67%, respectively.

**Table 3A:** Probability of target attainment (PTA) and cumulative fraction of response (CFR) of CZA/AZA against bloodstream infections (BSIs) caused by XDR/PDR *P. aeruginosa.*

Antibiotics	Dosing regimens	PTA of different MICs										CFR (%)	
		0.25	0.5	1	2	4	8	16	32	64	>64		
CZA	2.5g q8h	100	100	100	100	100	100	100	100	88.6	0	71.36	
	2.5g q6h	100	100	100	100	100	100	100	100	100	5.48	75.72	
	4g q8h	100	100	100	100	100	100	100	100	100	2.78	75.08	
	4g q6h	100	100	100	100	100	100	100	100	100	88.85	**95.63**	
	1.25g (0.5h) +1.25g (2h) q8h	100	100	100	100	100	100	100	100	100	**93.21**	**96.67**	
	1.25g (0.5h) +1.25g (2h) q6h	100	100	100	100	100	100	100	100	100	**96.05**	**97.35**	
	0.675g (0.5h) +0.675g (2h) q8h	100	100	100	100	100	100	100	100	100	80.34	**93.60**	
	0.675g (0.5h) +0.675g (2h) q6h	100	100	100	100	100	100	100	100	100	80.03	**93.53**	
AZA	2.5g q8h	100	100	100	100	100	100	100	100	89.49	0	71.59	
	2.5g q6h	100	100	100	100	100	100	100	100	100	75.75	**92.50**	
	4g q8h	100	100	100	100	100	100	100	100	100	14.15	77.79	
	4g q6h	100	100	100	100	100	100	100	100	100	**91.78**	**96.36**	
	1.25g (0.5h) +1.25g (2h) q8h	100	100	100	100	100	100	100	100	100	**94.35**	**96.95**	
	1.25g (0.5h) +1.25g (2h) q6h	100	100	100	100	100	100	100	100	100	**99.77**	**98.25**	
	0.675g (0.5h) +0.675g (2h) q8h	100	100	100	100	100	100	100	100	93.77	7.43	74.51	
	0.675g (0.5h) +0.675g (2h) q6h	100	100	100	100	100	100	100	100	99.83	14.67	77.87	

#### 3.4.2 Probability of target attainment of LRTIs

The PTA of each simulated dosing regimen for LRTIs was listed in **Table 3B**. All dosing regimens of CZA/AZA achieved a PTA of 100% when the MIC was ≤8 mg/L. When the MIC was 16 mg/L, CZA 2.5g q8h declined a PTA of 12.31%, and AZA 2.5g q8h declined a PTA of 31.95%. All OATA dosing regimens of CZA achieved a PTA of 100% when the MIC was 32 mg/L. When the MIC was 32 mg/L, AZA 2.5g q8h, 2.5g q6h, 4g q8h, 4g q6h, 1.25g (0.5h) +1.25g (2h) q8h, 1.25g (0.5h) +1.25g (2h) q6h, 2g (0.5h) +2g (2h) q8h, 2g (0.5h) +2g (2h) q8h, 2g (0.5h) +2g (2h) q6h achieved a PTA of 0, 0, 0.96%, 99.72%, 89.14%, 98.66%, 100% and 100%, respectively. When the MIC was 64 mg/L, CZA 1.25g (0.5h) +1.25g (2h) q8h, 1.25g (0.5h) +1.25g (2h) q6h, 2g (0.5h) +2g (2h) q8h, 2g (0.5h) +2g (2h) q6h achieved a PTA of 40.85%, 45.54%, 100% and 100%, respectively. When the MIC was 64 mg/L, AZA 2g (0.5h) +2g (2h) q8h, 2g (0.5h) +2g (2h) q6h achieved a PTA of 90.95% and 98.6%, respectively. When the MIC was >64 mg/L, CZA 2g (0.5h) +2g (2h) q8h, 2g (0.5h) +2g (2h) q6h achieved a PTA of 41.33% and 46.32%, respectively.

**Table 3B:** Probability of target attainment (PTA) and cumulative fraction of response (CFR) of CZA/AZA against lower respiratory tract infections (LRTIs) caused by XDR/PDR *P. aeruginosa.*

Antibiotics	Dosing regimens	PTA of different MICs										CFR (%)
		0.25	0.5	1	2	4	8	16	32	64	>64	
CZA	2.5g q8h	100	100	100	100	100	100	12.31	0	0	0	19.38
	2.5g q6h	100	100	100	100	100	100	100	0	0	0	31.16
	4g q8h	100	100	100	100	100	100	100	0	0	0	31.16
	4g q6h	100	100	100	100	100	100	100	0.13	0	0	44.61
	1.25g (0.5h) +1.25g (2h) q8h	100	100	100	100	100	100	100	100	40.85	0	58.53
	1.25g (0.5h) +1.25g (2h) q6h	100	100	100	100	100	100	100	100	45.54	0	59.79
	2g (0.5h) +2g (2h) q8h	100	100	100	100	100	100	100	100	100	41.33	84.29
	2g (0.5h) +2g (2h) q6h	100	100	100	100	100	100	100	100	100	46.32	85.48
AZA	2.5g q8h	100	100	100	100	100	100	31.95	0	0	0	23.68
	2.5g q6h	100	100	100	100	100	100	100	0	0	0	32.82
	4g q8h	100	100	100	100	100	100	100	0.96	0	0	33.00
	4g q6h	100	100	100	100	100	100	100	99.72	0	0	52.16
	1.25g (0.5h) +1.25g (2h) q8h	100	100	100	100	100	100	100	89.14	0	0	50.11
	1.25g (0.5h) +1.25g (2h) q6h	100	100	100	100	100	100	100	98.66	0	0	51.96
	2g (0.5h) +2g (2h) q8h	100	100	100	100	100	100	100	100	90.95	0	84.78
	2g (0.5h) +2g (2h) q6h	100	100	100	100	100	100	100	100	98.60	0	87.51

### 3.5 Cumulative fraction of response

#### 3.5.1 Cumulative fraction of response of BSIs

The cumulative fraction of response (CFR) of each simulated dosing regimen for BSIs was listed in **Table 3A**. If CZA was administered *via* traditional infusion, the CFR was 95.63% for 4g q6h. If CZA was administered *via* OTAT, the CFR was 96.67% for 1.25g (0.5h) +1.25g (2h) q8h, 97.35% for 1.25g (0.5h) +1.25g (2h) q6h, 93.60% for 0.675g (0.5h) +0.675g (2h) q8h and 93.53% for 0.675g (0.5h) +0.675g (2h) q8h. If AZA was administered *via* traditional infusion, the CFR was 92.50% for 2.5g q6h and 96.36% for 4g q6h. If AZA was administered *via* OTAT, the CFR was 96.95% for 1.25g (0.5h) +1.25g (2h) q8h, 98.25% for 1.25g (0.5h) +1.25g (2h) q6h. The above dosing regimens are considered to provide adequate PD exposures for the treatment of CZA/AZA against *XDR/PDR P. aeruginosa* BSIs.

#### 3.5.2 Cumulative fraction of response of LRTIs

**Table 3B** showed CFRs of CZA/AZA against LRTIs caused by XDR/PDR *P. aeruginosa*. CFRs were less than 90% for all simulated dosing regimens (i.e., 2.5 g [e.g., 1.25 g (0.5h) + 1.25 g (2h)] q6h, 2.5 g [e.g., 1.25 g (0.5h) + 1.25 g (2h)] q8h, 4g [e.g., 2 g (0.5h) + 2 g (2h)] q6h, 4g [e.g., 2 g (0.5h) + 2 g (2h)] q8h).

## 4 Discussion

In recent years, *P. aeruginosa* has spread widely worldwide and the treatment of BSIs or LRTIs caused by XDR/PDR *P. aeruginosa* has become a tough problem (Horcajada et al., 2019). The International Network for Optimal Resistance Monitoring Program (INORMP) in the United States (2012-2015) showed the prevalence of MDR and XDR *P. aeruginosa*, with rates of 15.4% and 9.4%, respectively (Sader et al., 2017b). Nowadays, CZA and AZA are considered treatment options for MDR/XDR *P. aeruginosa* infections (Horcajada et al., 2019). Besides, optimizing the use of antimicrobials that are currently available can be considered as a solution to this dilemma.

In this study, we collected 67 XDR/PDR *P. aeruginosa* isolates from a 3000-bed teaching hospital in northern China. Firstly, the MICs of CZA/AZA for XDR/PDR *P. aeruginosa* isolates were evaluated using the doubling dilution method. Secondly, we conducted WGS and performed bioinformatics analysis to determine the resistance genes of each isolate. Finally, we used MCS to analyze the PTA and CFR of different CZA/AZA dosing regimens.

We found that the resistance rate of CZA against XDR *P. aeruginosa* was 60%. *Schaumburg et al.* found that the resistance rate of CZA against XDR *P. aeruginosa* was 50.9% (Schaumburg et al., 2019). However, INORMP in the United States (2012-2015) showed that the sensitivity rate of CZA against XDR *P. aeruginosa* was 75.8% (Sader et al., 2017b). The different sensitivity rates of CZA against XDR *P. aeruginosa* may be related to the different sources and resistance genes of strains. In our study, all isolates were collected from northern China and most of the isolates produced OXA- β-lactamases. AVI has been shown in previous studies to be effective against OXA-48-producing isolates, but its effect on other OXA-β-lactams is unknown (Bhatnagar et al., 2021). This may explain the high resistance rate of CZA against the strains we collected.

Our work found that AZA was more effective than CZA for the treatment of MBL-producing XDR/PDR *P. aeruginosa* (e.g.: IMP-45, VIM-1, VIM-2). AVI is ineffective against MBL-producing isolates. However, the combination of AVI and ATM was a treatment option for MBL-producing isolates. *Lee et al.* also found that the combination of ATM and CZA may be a treatment option for VIM-2-producing *P. aeruginosa* (Lee et al., 2021). Our study found that CZA with avibactam at 8 and 16 mg/L was inactive against MBL-positive isolates. We also found that the *in vitro* activity of CZA/AZA against XDR/PDR *P. aeruginosa* could be improved with increasing AVI concentration. Nevertheless, *Yu et al.* found that CZA with avibactam at 8 and 16 mg/L was active against MBL-positive isolates (Yu et al., 2021). This may be because our collected strains also produced OXA-β- lactamases. Therefore, more exploration is needed in the future to figure out the resistance mechanism of XDR/PDR *P. aeruginosa*.

Our study had several interesting findings. Using whole genome sequencing, we found that XDR/PDR *P. aeruginosa* may carry some rare MBLs (e.g.: IND-6, SLB-1, THIN-B). IND-6 is a highly divergent IND-type MBL. It was first isolated from *Chryseobacterium indologenes* strain 597 in Burkina Faso (Zeba et al., 2009). SLB-1 was first identified from *Shewanella livingstonensis* in 2005 (Poirel et al., 2005). Besides, THIN-B was first identified from *Janthinobacterium lividum* (Rossolini et al., 2001). The resistance mechanism of XDR/PDR *P. aeruginosa* carrying IND-6, SLB-1, or THIN-B is needed to explore in the future.

The treatment of XDR/PDR *P. aeruginosa* infections was difficult, especially in immunocompromised patients (i.e.: patients who received hematopoietic stem cell transplantation, patients with nephrotic syndrome, patients with various malignant tumors) (Poole, 2011). Besides, *P. aeruginosa* has a huge intrinsic resistome and can be resistant to antibiotics through chromosomal mutations (Lister et al., 2009). Mobile genetic elements can be shared between *P. aeruginosa*. These elements produce carbapenemase enzymes, which makes *P. aeruginosa* resistant to carbapenems. These reasons have led researchers to develop novel antibiotics and methods to improve the therapeutic effect of XDR/PDR *P. aeruginosa* infections (Subedi et al., 2018). Our work found that OTAT could improve the PTA and CFR of CZA/AZA monotherapy for the treatment of critically ill patients with BSIs caused by XDR/PDR *P. aeruginosa*. From a pharmacoeconomic point of view, OTAT can reduce the financial burden of critically ill patients. The study by *Eguchi et al*. also confirmed that CFRs of OTAT with sufficient pharmacokinetic exposures were higher than traditional infusion (Eguchi et al., 2010). Besides, both OTAT and TI dosing regimens had poor efficacy against XDR *P. aeruginosa* LRTIs. This may be related to the lower penetration of ceftazidime-avibactam in the epithelial lining fluid compared to the blood (Nicolau et al., 2015).

Our study had several limitations that should be noted. Firstly, the collection of *P. aeruginosa* was confined to a small sample size and northern China. Secondly, our study only focused on partial beta-lactamases (class B β-lactamases and class D β-lactamases). Therefore, large-scale animal or clinical trials are needed in the future to confirm the efficacy of CZA/AZA against BSIs caused by XDR/PDR *P. aeruginosa*.

The main resistance mechanisms in *P. aeruginosa* are intrinsic, mutational, and horizontally acquired resistomes (Horcajada et al., 2019). We found that the efflux pump is indeed what makes *P. aeruginosa* drug resistant. The efflux pump was also considered in the study of this article (see the whole genome sequencing results in the **supplementary file** for details), but the efflux pump is not the focus of this article.

In conclusion, our work has the following results. Firstly, AZA was considered as an option for the treatment of XDR/PDR *P. aeruginosa* harbouring IMP-45, VIM-1, or VIM-2. Secondly, OTAT with sufficient PD exposure may be an optimal treatment option for BSI caused by XDR/PDR *P. aeruginosa* with a high-level MIC of CZA/AZA.

## Data availability statement

The original contributions presented in the study are publicly available. This data has been deposited into the NCBI repository under accession: PRJNA967114.

## Ethics statement

Ethical approval was granted by the Chinese People’s Liberation Army General Hospital.

## Author contributions

YK and JC designed and managed the project. YK performed all the experiments and wrote the manuscript. LX analyzed bioinformatics analysis. JY provided technical guidance. All authors contributed to the article and approved the submitted version.
